# Sexual attitudes, sexual behaviors, and use of HIV prevention services among male undergraduate students in Hunan, China: a cross-sectional survey

**DOI:** 10.1186/s12889-019-6570-2

**Published:** 2019-02-28

**Authors:** Hehua Xu, Jiaying Xie, Zhizi Xiao, Hong Xiao, Xianhong Li, Lloyd Goldsamt, Ann Bartley Williams, Honghong Wang

**Affiliations:** 1 Xiangya School of Nursing, Central South University, Changsha, 410013 Hunan China; 2 Department of Global Health, University of Washington, Seattle, WA USA; 3 New York University, New York, 10010 USA; 4 Xiangya School of Nursing, Central South University, 172 Tong Zi Po Road, Changsha, 410013 Hunan China

**Keywords:** Male undergraduate student, Sexual attitude, Sexual behaviour, HIV knowledge, HIV prevention service, China

## Abstract

**Background:**

The dramatic increase in human immunodeficiency virus (HIV) infection among undergraduate students in China, especially among the male students, is alarming. This study aimed to describe sexual attitudes and behaviours and to examine the use of HIV prevention services and related factors among male undergraduate students in Hunan, China.

**Methods:**

A cross-sectional survey was conducted from November 2017 to January 2018 among male students from three universities in Hunan, China. Self-administered questionnaires were uploaded online to collect data anonymously. HIV-related knowledge and sexual attitudes were assessed with the unified National AIDS Sentinel Surveillance Questionnaire and Sexual Attitude Scale, whereas sexual behaviours and use of HIV prevention services were examined with researcher-created questionnaires. HIV-related knowledge, sexual attitudes and behaviours, and use of HIV prevention services were described. Chi-square test and logistic regression were used to analyse the factors associated with the use of HIV prevention education services. *P* values ≤0.05 were considered significant.

**Results:**

Of the 1431 respondents, 1068 (74.6%; 95% CI: 72.4–76.0%) used HIV prevention education services and 105 (7.3%) took HIV testing. The openness of sexual attitudes was moderate overall. About 299 (20.9%) of this sample had active sex partners, and 49 (16.4%) of them had sex with males. The consistent use of condoms was unsatisfactory among the sexually active students, especially among those with homosexual behaviours. Participants who were older in age (OR: 0.77, 95% CI: 0.70–0.86), who were university seniors (OR: 0.80, 95% CI: 0.70–0.91), who drank alcohol (OR: 0.71, 95% CI: 0.55–0.93), and who had open attitude towards paid sex (OR: 0.72, 95% CI: 0.54–0.95), were less likely to use HIV prevention education services.

**Conclusions:**

Although male undergraduate students show open attitude to premarital sex and engage in risky sexual behaviours, their use of HIV prevention education services is unsatisfactory, particularly in terms of HIV testing. More comprehensive and specific education on HIV prevention and testing services should be designed and offered on campus.

## Background

The spread of human immunodeficiency virus (HIV) remains a critical global public health problem. According to statistics from the Joint United Nations Program on HIV/AIDS (UNAIDS), 36.9 million people worldwide were living with HIV in 2017 [1]. Sexual transmission is the most common mode of HIV transmission. Young adults, as a sexually active group, are especially susceptible to HIV infection. The estimated number of young adults (aged 15–24 years) newly infected with HIV in 2017 was 0.53 million, accounting for 29% of new HIV infections [1]. China had more than 0.79 million HIV-infected people as of April, 2018 [2]. Moreover, a striking increase in HIV prevalence among university students in China has been noted. Statistics from the Chinese Centre for Disease Control and Prevention (China CDC) revealed that from 2011 to 2015, the number of new HIV infections among university students increased by 35% annually and that male students accounted for 80% of the infections [3].

The epidemic could be attributed to several causes. Compared with older generations, contemporary Chinese university students are more open to premarital sex and more likely to begin sexual exploration in college [4]. Male university students possess more permissive attitude towards casual and premarital sexual activities than their female counterparts [5]. However, due to the lack of sexual health education, university students may have a higher likelihood of engaging in risky sexual behaviours, including having sex with commercial sex workers and/or multiple sex partners, and inconsistent use of condoms with casual sex partners [6, 7].

The rapid increase in HIV prevalence among university students has gained the attention of the Chinese government. The Ministry of Education and the Ministry of Health jointly issued a document on HIV prevention and among undergraduate students and required schools to provide mandatory education programs on HIV prevention starting in 2015 [8]. HIV prevention services for students are also widely promoted on campuses [9, 10]. Various activities organized by the colleges/universities have helped students improve their knowledge of HIV, sexual health, and HIV testing [11].

Despite better access to information on HIV prevention, university students failed to take full advantage of HIV prevention services and demonstrated inadequate knowledge of and improper behaviours in HIV prevention. According to previous studies, only 56.3% of students mastered the basic knowledge of HIV in Wuhan [12]. About 70% of the students still ignored consistent condom use, though they clearly knew that condom use could greatly reduce HIV risks [13]. The lack of translation from knowledge to prevention action was particularly severe among male university students [14, 15]. Although university students could access HIV testing through the Red Ribbon Association (a community-based organisation for HIV prevention in China) and blood donation vehicles as well as through self-testing kits available for purchase, only 4.5% of male university students had HIV testing in Guangzhou [16]. This number is even more sobering when compared with corresponding data from the United States, where 37.4% male university students received HIV testing [17]. Factors including low awareness and accessibility to testing services might impede the use of HIV-testing services among Chinese university students [18].

Many studies have described HIV-related knowledge, attitudes, and behaviours (including condom use) and HIV testing among college students [12–16], while information on the use of HIV prevention services, including HIV prevention education and HIV counselling and testing services is very limited but extremely important. It is urgent to conduct studies targeting male students, since HIV prevalence among undergraduate students has been growing at an alarming rate in the past five years, especially among male undergraduate students in China. The present study aims to describe the use of HIV prevention services, HIV-related knowledge, sexual attitudes and behaviours among male undergraduate students in Hunan, China, and to determine factors associated with the use of HIV prevention services. This study may collect information and provide evidence for the design of HIV prevention programs tailored to the preferences and characteristics of male undergraduate Chinese students.

## Methods

### Settings and participants

This cross-sectional study was conducted in Hunan, a Central China province. Home to 70 million people, Hunan has approximately 20,000 HIV-infected patients [19]. The 109 universities and colleges in Hunan provide higher education with a variety of disciplines. According to the document on HIV prevention jointly issued by the Ministry of Education and Ministry of Health, all colleges and universities are required to provide HIV/AIDS prevention education at the very beginning of university life [8]. To fully mobilise students’ enthusiasm in participating in AIDS prevention, various youth-friendly services such as lectures, billboards, videos, and school media publicity are encouraged in the universities. All universities/colleges in Hunan offer health education on AIDS prevention, but HIV testing is not available on university campus. Students can access HIV testing at local CDCs, nearby health facilities, or community-based organisations for HIV counselling and testing.

The target population of this study was male undergraduate students. Approximately 60,000 male undergraduate students from three comprehensive universities in Hunan were purposefully sampled to ensure an adequate representation of students from various majors and different grades. With the permission of the Student Union of the three universities and departments, we asked for recruitment assistance from each grade. The participants were recruited via peer referral in the selected cohorts. Via WeChat (a Chinese cell/web app for messaging, social media, communications – including answering questionnaires – and other purposes), we successfully sent a unique two-dimensional code linking the electronic questionnaire to potential participants who worked for the student unions of various grades in the universities. In all three universities, these participants then disseminated the two-dimensional code via WeChat group to undergraduates of different grades and various majors. Male undergraduate students who were 18 years and above and used WeChat were eligible for this study*.* The sample size was calculated based on the proportion of HIV testing. The rate of HIV testing reported in previous studies in Chinese undergraduate students varied from 2.8–47.1% [20, 21]. Based on the detection table of the sample size for proportions in Hulley et al. [22], assuming the prevalence of HIV testing was 25%, and for a confidence level of 95% with the width of confidence interval of 0.10, the optimum estimate of 288 students was required. As sexual experience is a precondition of HIV testing, the total sample size based on the proportion of sexual behavior occurrence among Chinese male undergraduate students (21.1%) [23] was 1365 by assumption in this study.

### Study variables and measurements

Demographic data regarding the students’ age, grade, ethnicity, area of residence, with or without a sexual partner, etc. were collected. Behaviours such as smoking, alcohol drinking, and sexual orientation were also assessed.

HIV-related knowledge was assessed with a 13-item questionnaire. The questionnaire was developed on the basis of the unified National AIDS Sentinel Surveillance Questionnaire designed specifically for university students [24] and the existing literatures [25]. Each item was measured via a *yes/no/uncertain* format, and only the correct response scored 1 point. Mastering 85% or more of the knowledge in the questionnaire was considered as having sufficient knowledge, and a total score of 11 or higher was required to demonstrate ‘sufficient knowledge’; otherwise, respondents were regarded as having ‘insufficient knowledge’. The internal reliability (KR-20) for this questionnaire was 0.64.

In this study, the Sexual Attitude Scale was used to measure the undergraduates’ sexual attitudes [26]. The 7-item scale assessed the attitudes towards premarital sex, extramarital sex, commercial sex, homosexual behaviours, one-night stand, and multiple sex partners. Participants rated items on a Likert scale ranging from 1 (completely unacceptable) to 5 (completely acceptable). For each item, scores lower than 3 were recoded as ‘unacceptable’, and scores of 3 and higher were recoded as ‘acceptable’. A composite score was obtained by summing up the scores of each item, with higher scores indicating more conservative sexual attitudes. The original 6-item scale assessed commercial sex behavious without distinguishing between ‘paying for sex’ and ‘selling sex’. In the current study, participants were asked to respond to both situations. The Cronbach’s alpha for this scale was 0.84.

Sexual behaviours were assessed by asking students about their sexual experiences, heterosexual/ homosexual behaviours, commercial sexual behaviours, and condom use in the past year [24]. Participants who had sexual intercourse in the last year were asked to rate the frequency of condom use on the basis of four response options (1 = never, 2 = sometimes, 3 = often, 4 = always). HIV prevention services were operationally defined in this study as HIV educational programs, counselling services, and testing services. Considering the differences between the HIV prevention services provided by colleges and universities, public health sectors, and community-based organisations, the following five questions were asked: (1) *Did you receive any HIV prevention education organised by the university in the last year?* (2) *What types of educational activities did you participate in at the university?* (3) *Did you take an HIV test in the last year?* (4) *Are you willing to take an HIV test?* and (5) *If you are not willing to take an HIV test, what are your reasons for refusing to do so?* These questions were designed by the investigators after collecting information from people who organized campus HIV prevention programs and after conducting literature reviews [27, 28].

### Data collection

Data were collected online via the Chinese professional survey website *Wenjuanxing* (www.sojump.com) from November 2017 to January 2018. All potential participants accessed the questionnaires by scanning the two-dimensional code with WeChat, and they were able to submit the questionnaires only when all items were finished. Students were allowed to complete the self-administered questionnaire, which took approximately 20–30 min, in their spare time. Each participant received a bonus of 5 RMB online upon the completion of the survey. To avoid repetition, questionnaires bearing the same IP address as previously submitted questionnaires were not accepted.

### Statistical analysis

The data were analysed with SPSS version 20.0. Frequencies, percentages, means, and standard deviations were used to describe the demographic variables, HIV-related knowledge, sexual attitudes and behaviours, and use of HIV prevention services. A chi-square test was used to assess the association between sexual behaviours and the frequency of condom use. Additionally, univariate and multivariate logistic regression analyses were conducted to examine factors associated with the use of HIV prevention education services in colleges and universities. Variables identified as statistically significant at a level of *p* <  0.1 in the univariate logistic regression analysis were entered into the multivariate logistic regression model with a stepwise forward approach. All statistical analyses were two-tailed, and α level of 0.05 denoted statistical significance.

## Results

### Demographic characteristics

Of the 2432 invited male undergraduate students, 254 were excluded by the inclusion criteria (56 were under 18 years old and 198 were females). Of the remaining 2178 students, 747 (34.3%) declined to complete the questionnaire, resulting in a final sample of 1431 completed and valid questionnaires. The participants were 18 to 25 years (mean = 19.5 years; SD = 1.32). Most were of Han nationality (93.6%) from urban districts (56.3%), majoring in nonmedical fields (94.9%), not living with sexual partners (96.7%), and from lower grades (27.9% freshmen and 33.3% sophomores). Roughly one-fifth of the participants self-reported cigarette smoking, and more than half self-reported alcohol drinking. The majority of the students regarded themselves as heterosexual (87.8%), and the remaining students considered themselves as either homosexual, bisexual, or unclear (12.2%) (Table 1).

**Table 1:** Demographic data of the male undergraduate students (*N* = 1431)

Item	Category	Number	Percent
Field of study	Medical	73	5.1
	Nonmedical	1359	94.9
Living with sexual partner	Yes	47	3.3
	No	1384	96.7
Ethnicity	Han	1340	93.6
	Others	91	6.4
Grade	Freshmen	399	27.9
	Sophomore	477	33.3
	Junior	296	20.7
	Senior	259	18.1
Area of residence	Urban	806	56.3
	Rural	625	43.7
Sexual orientation	Heterosexual	1256	87.8
	Homosexual	59	4.1
	Bisexual	56	3.9
	Unclear	60	4.2
Cigarette smoking	Yes	283	19.8
	No	1148	80.2
Alcohol drinking	Yes	925	64.6
	No	506	35.4

### HIV knowledge

Overall, 1099 (76.8%; 95% CI: 74.6–79.0%) students scored 11 or above in HIV-related knowledge, and were considered as having sufficient knowledge. Among the 13 HIV knowledge questions, the two items on safe sex were answered correctly mostly: 97.7% of the students believed *unprotected sex would increase the probability of HIV infection*, and 95.7% thought *using condoms during sex would decrease HIV transmission*. Four items had a correct response rate below 80%: two items on HIV transmission routes, namely *whether one could be infected through mosquito bites* and *whether one could be infected by kissing HIV-positive people*, showing a correct response rate of 75.3 and 69.7% respectively. The other two items related to the situation after HIV infection had the lowest correct response rate of 69.0 and 62.9%, namely whether *AIDS was equal to death* and whether *HIV antibody could be detected within one week after the infection*. These answers indicated that students with sufficient HIV knowledge still had misconceptions that needed further clarification.

### Sexual attitudes and behaviours

Our sample demonstrated a highly acceptable attitude towards premarital sex and a neutral attitude towards homosexual sex. For premarital sex, 77.1% of the participants reported acceptable (median = 3, P_25_ = 3, P_75_ = 5), and 41.8% reported homosexuality acceptable (median = 2, P_25_ = 1, P_75_ = 3). As to other types of sexual behaviours, 27.9% believed one-night stand acceptable (median = 2, P_25_ = 1, P_75_ = 3), 22.8% expressed paying for sex acceptable (median = 1, P_25_ = 1, P_75_ = 2), 17.3% considered selling sex acceptable (median = 1, P_25_ = 1, P_75_ = 2), 15.2% thought having multiple sexual partners acceptable (median = 1, P_25_ = 1, P_75_ = 2), and 13.2% stated extramarital sex acceptable (median = 1, P_25_ = 1, P_75_ = 2).

Totally 299 out of the 1431 male undergraduate students (20.9%; 95% CI: 18.8–23.0%) reported active sex partners, and 49 out of the 299 (16.4%/) engaged in sexual behaviours with males. Regarding sexual partners in the last year among the 299 sexually actively students, 250 (83.6%) reported having sex with fixed female partners, 81 (27.1%) had casual female partners (81 having both fixed and casual female partners), and 15 (5%) had commercial female partners; whereas 33 (11%) had fixed male partners, 22 (7.4%) had casual male partners (20 having both fixed and casual male partners), and 11 (3.7%) had commercial male partners. Those students who had sex with females (*t* = 3.865, *p* <  0.001) or male casual partners (*t* = 2.257, *p* = 0.029) scored higher in sexual attitudes with more open sexual attitudes. Frequencies of condom use were significantly different among the groups with different partners ( χ^2^ = 17.016, *p* = 0.048). The post hoc analysis showed that the variance was mostly attributable to the larger proportion of students who never used condoms when they had sex with casual or fixed male partners and a smaller percentage to those with fixed female partners (*p* < 0.05) (Table 2).

**Table 2:** Frequency of condom use during sex with different types of partners in the past year (*n* = 299)

Sexual behaviour	n	Frequency of condom use, n (%)			
		Never	Sometimes	Often	Always
Hetero-sex with fixed partners	250	40 (16.0)	37 (14.8)	61 (24.4)	112 (44.8)
Hetero-sex with casual partners	81	16 (19.8)	14 (17.3)	13 (16.0)	38 (46.9)
Homo-sex with fixed partners	33	12 (36.4)	4 (12.1)	6 (18.2)	11 (33.3)
Homo-sex with casual partners	22	9 (40.9)	1 (4.5)	4 (18.2)	8 (36.4)

### Use of HIV prevention services

Of all the male undergraduate students in the sample, 1068 (74.6%; 95% CI: 72.4–76.0%) used HIV prevention education services provided by colleges universities in the last year. More than half of the students had used two forms of the university education services, including publicity materials / billboards and lectures. Other educational services included HIV testing services. Only 105 (7.3%) male students had HIV testing in the last year, but the majority (1110, 77.6%) reported willingness to take the tests, and the reasons of 321 students’ unwillingness to take HIV testing were given (Table 3).

**Table 3:** Use of HIV prevention services in the past year

Service	Number of students, n (%)
HIV prevention services in universities	
Providing publicity materials or billboards	723 (67.7%)
Delivering lectures	667 (62.5%)
Organising volunteers to host propaganda campaigns	500 (46.8%)
Providing information via new school media	440 (41.2%)
Playing HIV-related videos	214 (20.0%)
Conducting debates	117 (10.9%)
Hosting artistic performances	73 (6.8%)
Others	13 (1.2%)
HIV testing	
Had HIV testing before	105 (7.3%)
Willingness to take HIV testing	1110 (77.6%)
Reasons for unwillingness to take HIV testing	
Firm belief in non-HIV infection	235 (73.2%)
No idea of HIV testing sites	46 (14.3%)
Escape the anxiety of HIV positive result	45 (14.0%)
Never heard of HIV/AIDS	22 (6.9%)
Belief in unlikely infection with high-risk sex only occasionally	15 (4.7%)
Others	22 (6.9%)

### Factors related to the use of HIV prevention education services in colleges and universities

Variables significant at *p* <  0.1 level were included in the multivariate logistic regression. Students who were older (OR: 0.77, 95% CI: 0.70–0.86), from higher grade (OR: 0.80, 95% CI: 0.70–0.91), and those who drank alcohol (OR: 0.71, 95% CI: 0.55–0.93), and had more open attitudes towards paying for sex (OR: 0.72, 95% CI: 0.54–0.95) were less likely to use HIV prevention education services in colleges and universities (Table 4).

**Table 4:** Factors associated with the use of HIV prevention services in universities

Variable	Category	Univariate logistic regression		Multivariate Logistic regression	
		OR (95% CI)	*p*	OR (95% CI)	*p*
Age	Per advanced year	0.71 (0.65, 0.77)	< 0.001	0.77 (0.70, 0.86)	< 0.001
Field of study	Nonmedical	Ref			
	Medical	1.33 (0.75, 2.38)	0.333	–	
Living with sexual partner	No	Ref			
	Yes	0.72 (0.38, 1.34)	0.296	–	
Ethnicity	Han	1.12 (0.70, 1.81)	0.633	–	
	Others	Ref			
Grade	Per advanced grade	0.68 (0.61, 0.76)	< 0.001	0.80 (0.70, 0.91)	0.001
Area of residence	Urban	Ref			
	Rural	0.79 (0.63, 1.01)	0.076	NS	
Sexual orientation			0.997		
	Heterosexual	1.01 (0.71, 1.47)		–	
	Homosexual	1.00 (0.55, 1.81)		–	
	Bisexual	0.84 (0.47, 1.53)		–	
	Unclear	Ref			
Cigarette smoking	Yes	0.71 (0.54, 0.95)	0.021	NS	
	No	Ref			
Alcohol drinking	Yes	0.69 (0.53, 0.89)	0.005	0.71 (0.55, 0.93)	0.012
	No	Ref			
HIV knowledge	Insufficient	Ref			
	Sufficient	1.4 (1.09, 1.87)	0.011	NS	
Attitude towards sex	Multiple sexual partners	1.07 (0.76, 1.49)	0.697		
	Selling sex	0.77 (0.57, 1.05)	0.100		
	Extramarital sex	1.10 (0.77, 1.58)	0.597		
	Paid sex	0.68 (0.52, 0.89)	0.005	0.72 (0.54, 0.95)	0.021
	One-night stand	0.82 (0.64, 1.07)	0.144		
	Homosexual behaviour	0.77 (0.61, 0.98)	0.033	NS	
	Premarital sex	0.92 (0.69, 1.23)	0.568		

*Ref* reference, *OR* odds ratio, *CI* confidence interval, *NS* not significant in the multivariate analysis, — variables were not included in the multivariate analysis

## Discussion

Due to the deep influence of Confucianism for thousands of years, Chinese people are believed to be relatively more conservative to sex topics and activities than people in western countries. Chinese greatly emphasize fidelity in marriage relations. However, the moderately open sexual attitudes to premarital and homosexual behaviours uncovered in this study showed the great change in sexual attitudes nowadays among male undergraduate students as compared with those of a decade ago [29, 30]. Risky sexual behaviours, especially among homosexual males, were common in this study. The use of HIV prevention services (HIV education at colleges and universities and HIV testing services) was not satisfactory in this sample.

Male undergraduate students in Hunan exhibited open attitudes to premarital sex and homosexuality, which has also been shown in previous studies [31, 32], indicating that premarital sex has became frequent. Male students’ acceptance of homosexuality was also shown by Kar and colleagues [33]. Although open sexual attitudes help decrease the level of concealment and stigma associated with homosexuality [33], such attitudes may result in an increase in high-risk sex. Sex education, especially education on safe sex, should be urgently incorporated into current HIV prevention programs on campuses.

Regarding sexual behaviours, about 21% of the male undergraduate students were sexually active with inconsistent use of condoms, and having sex with casual partners was also common. This finding echoes a previous study conducted in Hefei, China [34], but is much lower than that in Ethiopia reporting more than 70% of university students were sexually active [35]. Alarmingly, though unprotected sex was reported disproportionately by male students with different sexual orientations, it was more frequent during homosexual behaviours, in line with Rong’s study [36], which found that risky sexual behaviours were significantly more prevalent among homosexual and bisexual males than heterosexual males. Given the situation that pre-exposure prophylaxis is not widely accessible in China [37], HIV prevention programs specifically designed to reduce high-risk sexual behaviours among men having sex with men (MSM) are quite necessary on campuses.

Nearly four-fifths of this population demonstrated sufficient knowledge by the National AIDS Sentinel Surveillance Questionnaire to assess HIV knowledge. However, high level of HIV knowledge did not show significant associations with safe sex behaviours and use of HIV prevention services. The inconsistency between sufficient HIV knowledge and engaging in risky sex may be caused by the limited HIV knowledge acquired in China, which includes only basic HIV knowledge tailored to the general population. No knowledge about the details of sexual behaviours and unprotected sex was available in the curriculum for most Chinese college and university students. More comprehensive, specific, and detailed information on HIV, especially knowledge of safe sex, should be included in specific HIV knowledge questionnaires and school-based HIV education services.

According to this study, the use of the HIV education services in colleges and universities was only slightly higher than 70%, much lower than the expectation that all undergraduate students should receive HIV prevention education as part of the mandatory training [10]. One potential reason is that the formality of HIV education is too simple and homogenised. The main types of education consist of giving out flyers and pamphlets or delivering lectures by specialists, which are less attractive to students and not effective enough for behaviour changes. Furthermore, the present HIV prevention services on campuses mainly aim at spreading knowledge and information, and feedback from students are lacking. The related knowledge and information provided by colleges and universities are not specific enough for undergraduate students, and the tactics of conducting protected sexual behaviours are sometimes not included in the education.

As suggested in Chi’s study [38], HIV prevention education should be more specific to the targeted population, and diverse teaching techniques should be adopted and more comprehensive information included. Considering contemporary education services and the national regulation that HIV education in colleges and universities should clearly focus on students [39], it is essential that university HIV education services for male students include useful and practicable knowledge and details which should be distinguished from general HIV education services. Our findings indicated that male students who were older or in higher grades, and who drank alcohol and had more open attitudes towards paying for sex were less likely to utilise the HIV prevention services organised by universities, suggesting more integrated HIV prevention services are needed for these subgroups. Further qualitative studies are encouraged to help determine the specific needs of male undergraduates and better evaluate HIV education services in colleges and universities.

The extremely low use of HIV testing services was also worrisome. We found that the rate of accepting HIV testing was far lower than that of the willingness to be tested due to concerns about privacy exposure and the lack of routine HIV testing in the health service facilities on campuses [40], and also rapid HIV testing has not been widely adopted by colleges and universities in China. Two of the three universities included in this study did not offer rapid HIV testing among students during our survey. This sharply decreased the accessibility of HIV testing, partially contributing to the low rate of testing among this sample. Most of the concern focused on male students who are sexually active (particularly homosexually active), who engage in risky sexual behaviours, and who have inadequate access to individualised HIV prevention counselling and HIV testing on campus. They also have concerns about the potential burden of HIV-positive results which in turn contributes to the low use of HIV testing service [41]. Integrating HIV counselling and testing services into university health care services will help improve the accessibility of HIV services among students and meet the needs of those who are at high risk of HIV infection. Wu and colleagues’ [42] suggested that sharing and integrating resources are important strategies for AIDS prevention in colleges and universities. We also suggest that routines for providing HIV services should be developed. Moreover, students should be widely informed of the availability of these services and provided with sufficient contact resources.

Several limitations may influence the generalisability of the findings. Firstly our participants were recruited from only three universities in Hunan, and caution should be aroused as to the application of these findings to other university students. Secondly all the data were self-reported and might be subject to selection bias, measurement error, or desirability bias, especially the data on risky sex. Furthermore, the questionnaires for measuring sexual behaviours and use of HIV prevention services were devised by the researchers and thus validation should be taken into consideration. Future studies with more representative samples and more standardised and valid measurements are expected to provide important evidence for developing effective HIV services in Chinese colleges and universities for male undergraduate students.

## Conclusions

Although male undergraduate students demonstrate an open attitude to premarital sex and engage in high-risk sexual behaviours, their use of HIV prevention education services is unsatisfactory. HIV education services in Chinese colleges and universities should be designed and provided comprehensively taking into consideration the needs of male undergraduates, including open sexual attitudes, risky sex, imbalance between HIV knowledge and high-risk behaviours, and willingness to take HIV testing. Programs aiming to change the risky sexual behaviours and develop accessible HIV counselling and testing services on campuses are urgent and critical for HIV prevention in Chinese colleges and universities.

## Abbreviations

China CDC: the Chinese Centre for Disease Control and Prevention
HIV: Human immunodeficiency virus
MSM: Men having sex with men
UNAIDS: the Joint United Nations Program on HIV/AIDS
